# Computational thinking and academic imposter syndrome: a serial mediation model of creative and critical thinking

**DOI:** 10.3389/fpsyg.2026.1797656

**Published:** 2026-04-13

**Authors:** Adem Yilmaz, Mehmet Koray Serin

**Affiliations:** 1 Department of Mathematics and Science Education, Faculty of Education, Kastamonu University, Kastamonu, Türkiye; 2 Department of Primary Education, Faculty of Education, Kastamonu University, Kastamonu, Türkiye

**Keywords:** academic imposter syndrome, computational thinking, creative thinking, critical thinking, higher-order thinking, serial mediation

## Abstract

**Objective:**

This study aimed to examine the relationships among computational thinking, creative thinking, critical thinking, and academic imposter syndrome within a serial mediation framework. More specifically, it investigated whether creative thinking and critical thinking sequentially mediate the relationship between computational thinking and academic imposter syndrome among university students.

**Methods:**

A quantitative, cross-sectional research design was employed. Data were collected from 728 undergraduate students enrolled at public universities in Türkiye. Participants completed validated self-report instruments assessing computational thinking, creative thinking dispositions, critical thinking dispositions, and academic imposter syndrome. Preliminary analyses were conducted to evaluate the reliability and validity of the measurement instruments. To test the proposed model, serial mediation analyses were performed using Hayes’ PROCESS macro (Model 6) in SPSS. Bootstrap procedures with 5,000 resamples were used to estimate indirect effects and 95% confidence intervals.

**Results:**

Computational thinking was positively associated with both creative thinking and critical thinking, and creative thinking was also a significant positive predictor of critical thinking. In contrast, computational thinking, creative thinking, and critical thinking were all negatively associated with academic imposter syndrome. The serial mediation analyses further showed that creative thinking and critical thinking sequentially mediated the relationship between computational thinking and academic imposter syndrome. All indirect effects, including the total indirect effect, were statistically significant, as their confidence intervals did not include zero.

**Discussion:**

The findings indicate that computational thinking is associated with lower levels of academic imposter syndrome both directly and indirectly through creative and critical thinking. In this sense, higher-order cognitive skills may help explain more adaptive academic self-perceptions and lower levels of imposter-related feelings among university students. These results underscore the importance of integrating computational, creative, and critical thinking into higher education curricula to support both academic development and students’ emotional adjustment.

## 1 Introduction

Higher education increasingly requires students not only to acquire disciplinary knowledge but also to regulate their learning, evaluate their own performance, and respond effectively to complex cognitive demands (Hague, 2024; Lee et al., 2024; Lobos et al., 2024; OECD, 2019). In contemporary academic environments, students are expected to analyze information, solve multifaceted problems, adapt to new situations, and construct coherent academic identities while their performance is continuously assessed and compared (Pérez-Jorge et al., 2025). As a result, academic functioning is shaped not only by what students know, but also by how they think, interpret feedback, and judge their own competence (Anderson, 2015; Dörrenbächer-Ulrich et al., 2024; Hartelt and Martens, 2024). In this context, understanding the cognitive processes associated with academic self-perceptions has become an important issue in educational and cognitive science research. Among the self-perceptions that have attracted growing scholarly attention, academic imposter syndrome stands out as a particularly relevant phenomenon. Students experiencing imposter-related feelings often doubt their abilities, attribute their achievements to external factors, and fear being exposed as less competent than others perceive them to be (Dumitrescu and De Caluwé, 2024; Ménard and Chittle, 2023; Yang et al., 2024). Such experiences are associated with anxiety, reduced confidence, and maladaptive academic functioning, even among objectively capable or high-achieving individuals (Chua et al., 2025; El-Ashry et al., 2024). Because these perceptions are closely connected to how students process academic demands, evaluate their own performance, and regulate their learning, academic imposter syndrome may be understood not only as a psychological difficulty but also as a self-evaluative outcome rooted in broader cognitive processes. Among the broad range of higher-order thinking skills discussed in the literature, the present study focuses specifically on computational thinking, creative thinking, and critical thinking because these constructs represent complementary dimensions of complex cognition. Computational thinking reflects structured problem representation and procedural reasoning, creative thinking reflects flexible idea generation and novel solution production, and critical thinking reflects evaluative judgment and reflective analysis (Hemmler and Ifenthaler, 2024). Examining these skills together provides a more integrated perspective on how students process academic demands and construct judgments about their own competence. This integrated perspective is particularly relevant for understanding academic imposter syndrome, which is closely tied to self-evaluation, perceived competence, and the interpretation of academic performance. Against this background, the present study examines whether computational thinking is associated with academic imposter syndrome directly and indirectly through creative and critical thinking. By doing so, the study seeks to move beyond isolated examinations of individual variables and to develop a more coherent cognitive explanation for academic self-perceptions in higher education. It should be noted, however, that the present study does not assess cognitive performance through objective or task-based measures. Rather, it examines self-reported dispositions and perceptions related to computational, creative, and critical thinking in relation to academic imposter syndrome. Accordingly, the proposed model should be interpreted as reflecting perceived higher-order thinking tendencies and academic self-evaluations rather than directly observed cognitive performance or experimentally verified cognitive mechanisms.

### 1.1 Literature review

#### 1.1.1 Computational thinking

Computational thinking has gained increasing attention as a foundational cognitive skill in the digital age. Originally rooted in computer science, it was conceptualized by Wing (2006) as a broadly applicable problem-solving approach involving decomposition, abstraction, algorithmic reasoning, and systematic evaluation. Subsequent scholarship has further emphasized that computational thinking is not limited to programming contexts, but instead reflects a general cognitive framework that supports reasoning across academic domains (Denning, 2017; Ngadengon et al., 2024). Through processes such as identifying relevant information, recognizing patterns, constructing step-by-step procedures, and evaluating outcomes logically, computational thinking enables individuals to approach complex tasks in structured and efficient ways (Ngadengon et al., 2024; Ocampo et al., 2024). Empirical studies have shown that students with stronger computational thinking skills tend to demonstrate greater cognitive flexibility, enhanced analytical reasoning, and improved capacity for managing complex information (Montuori et al., 2024; Shute et al., 2017). Computational thinking also appears to support self-regulation and metacognitive awareness by encouraging learners to monitor their problem-solving processes and revise ineffective strategies when needed (Buwono et al., 2025; Choi and Bae, 2025; Kong et al., 2020; Rajadurai and Ganapathy, 2023; Yao and Lin, 2025). In this sense, computational thinking may be considered a foundational cognitive resource that supports other higher-order thinking processes relevant to academic functioning. However, although its role in problem solving and academic performance has been widely discussed, relatively limited attention has been devoted to how computational thinking may shape students’ academic self-perceptions and imposter-related experiences (Yang et al., 2024).

#### 1.1.2 Creative thinking

Creative thinking represents another essential dimension of higher-order cognition. It refers to the ability to generate novel, original, and useful ideas by combining existing knowledge in flexible and unconventional ways (Runco and Jaeger, 2012). As such, it involves divergent thinking, cognitive flexibility, and the capacity to explore multiple perspectives when addressing complex problems (Villalustre et al., 2024). From a cognitive science perspective, creative thinking emerges from the interaction of associative memory, executive control, and evaluative processes, allowing individuals to generate possibilities while simultaneously monitoring their relevance and feasibility (Ayyıldız and Yılmaz, 2021; Kenett, 2025; Tregenza et al., 2025). Within educational contexts, creative thinking plays a vital role in promoting deep learning, intellectual engagement, and adaptive academic behavior (Beghetto and Kaufman, 2014). Students with stronger creative thinking skills are more likely to approach academic tasks with curiosity, persistence, and openness to experimentation. They may also be better able to reframe difficulties, generate alternative strategies, and respond flexibly to uncertainty (Amabile, 1996; Karunarathne and Calma, 2024; Zaremohzzabieh et al., 2025). In the present study, creative thinking is considered not merely as an isolated disposition, but as a generative cognitive mechanism through which students may respond more adaptively to academic complexity. Its role is especially important because academic self-perceptions may depend not only on the ability to solve problems systematically, but also on the capacity to generate new ways of understanding and addressing challenges.

#### 1.1.3 Critical thinking

Critical thinking is widely recognized as a core component of higher-order cognitive processing. It encompasses the ability to analyze arguments, evaluate evidence, identify inconsistencies, and make reasoned judgments (Facione, 2015). This form of thinking is guided by standards such as clarity, relevance, coherence, and accuracy, and it depends on executive functions, working memory, and metacognitive monitoring (Jaramillo Gómez et al., 2025; Li et al., 2024). Individuals engaged in critical thinking must coordinate attention, inhibit irrelevant information, compare alternatives, and evaluate the strength of available evidence. These processes are fundamental for rational decision making and informed problem solving in academic settings (Arifin et al., 2025; Jaramillo Gómez et al., 2025). In higher education, critical thinking is often associated with academic success, effective self-regulation, and realistic self-assessment (Borbon et al., 2025; Rothinam et al., 2025). Students with stronger critical thinking skills are more capable of assessing the quality of information, resisting cognitive biases, and evaluating their own performance objectively. This makes critical thinking especially relevant to academic self-perceptions. Moreover, critical thinking complements creative thinking in important ways. Whereas creative thinking supports the generation of multiple possibilities, critical thinking supports the evaluation, refinement, and justification of those possibilities. Taken together, creative thinking and critical thinking may therefore be viewed as complementary dimensions of higher-order cognition, with one supporting idea generation and the other supporting reflective evaluation (Yang et al., 2025).

#### 1.1.4 Academic imposter syndrome

Against this backdrop of cognitive skill development, academic imposter syndrome has emerged as a significant phenomenon affecting students across disciplines. First identified by Clance and Imes (1978), imposter syndrome refers to persistent feelings of intellectual fraudulence, self-doubt, and fear of being exposed as incompetent despite objective evidence of competence. Individuals experiencing imposter syndrome tend to attribute their achievements to external factors such as luck or excessive effort rather than to their own ability (El-Ashry et al., 2024). These distorted attributions may undermine confidence and contribute to chronic anxiety, reduced motivation, and avoidance of challenging academic tasks (Chua et al., 2025). From a cognitive science perspective, academic imposter syndrome may be interpreted as a maladaptive self-evaluative outcome shaped by biased metacognitive judgments and distorted interpretations of performance (Emas et al., 2024). Students who struggle to organize, generate, and evaluate their thinking effectively may have greater difficulty integrating feedback, recognizing progress, and judging their own competence accurately (Newar et al., 2025; Silverman, 2024). In this sense, academic imposter syndrome does not simply reflect actual incompetence; rather, it reflects a misalignment between cognitive capacities and self-perceptions. This makes it a particularly relevant outcome variable in studies examining higher-order cognitive functioning and academic resilience (Slimi et al., 2024).

### 1.2 Theoretical integration and hypothesis development

The present study does not treat computational thinking, creative thinking, and critical thinking as unrelated cognitive traits; rather, it conceptualizes them as sequentially connected processes through which students interpret, manage, and evaluate academic demands. Computational thinking provides a structured foundation for approaching problems, organizing information, and generating systematic solutions. Creative thinking extends this process by enabling individuals to produce flexible and original responses to academic challenges. Critical thinking then supports the evaluation, refinement, and justification of these responses through reflective judgment and evidence-based reasoning. Considered together, these three forms of thinking represent a coherent higher-order cognitive system that may shape how students interpret their academic experiences and judge their own competence. Within this framework, the proposed serial ordering is theoretically grounded in the functional sequence of higher-order cognition. Computational thinking is positioned as an initial structuring process because it helps students define problems, organize information, and generate systematic pathways for action. Creative thinking is then conceptualized as a generative process through which multiple possibilities, alternatives, and interpretations can be produced on the basis of that structured cognitive groundwork. Critical thinking follows as an evaluative process, enabling students to assess, refine, and justify those possibilities through reflective judgment and evidence-based reasoning. Although alternative configurations may also be plausible, the present ordering was selected because it reflects a coherent progression from structured problem representation to idea generation and finally to evaluative regulation. This integrated perspective is especially important in the case of academic imposter syndrome. If students are able to process problems systematically, generate adaptive alternatives, and evaluate their performance more realistically, they may be less likely to develop distorted self-perceptions and persistent feelings of inadequacy. Conversely, weaknesses or disruptions in these interconnected cognitive processes may contribute to maladaptive self-evaluation and heightened vulnerability to imposter-related experiences (Alammar et al., 2025). In this way, computational thinking may influence academic self-perceptions not only directly but also indirectly through its effects on creative and critical thinking.

A serial mediation model is therefore theoretically plausible. Computational thinking may foster creative thinking by promoting structured exploration, flexible problem representation, and metacognitive awareness (Günaltay et al., 2025). In turn, creative thinking may contribute to critical thinking by encouraging individuals to generate multiple perspectives and then refine these perspectives through evaluative analysis (Runco, 2014; Shaber et al., 2025). Together, creative and critical thinking may function as cognitive mechanisms through which computational thinking contributes to more realistic and confident academic self-evaluations. Although prior studies have documented the educational value of these constructs, the literature still lacks a coherent model explaining how they operate together to shape academic imposter syndrome (Cheung and Cheng, 2024). Indeed, previous research has often examined computational thinking, creativity, and critical thinking as separate predictors of academic performance or general well-being (Montuori et al., 2024; Ngadengon et al., 2024; Ocampo et al., 2024; Shute et al., 2017). Likewise, research on imposter syndrome has predominantly focused on personality traits, social influences, and demographic characteristics, with comparatively limited attention to the underlying cognitive processes that may protect against or contribute to imposter experiences (Chua et al., 2025; El-Ashry et al., 2024; Smith, 2025; Yang et al., 2024). This gap limits both theory and practice, because interventions that target confidence alone may remain insufficient if they do not address the cognitive mechanisms through which students form self-evaluations (Hsu et al., 2024; Jansson et al., 2025; Para et al., 2024).

### 1.3 Present study and hypotheses

Accordingly, the present study seeks to contribute to the literature by proposing and testing a serial mediation model in which creative thinking and critical thinking mediate the relationship between computational thinking and academic imposter syndrome. Grounded in cognitive science theory, this model conceptualizes academic self-perceptions as outcomes of interconnected information-processing and evaluative systems. By adopting this perspective, the study moves beyond simple association analyses and offers a more nuanced account of the mechanisms through which cognitive skills may shape psychological experiences in higher education. Specifically, this study addresses the following research problem: To what extent does computational thinking explain variations in academic imposter syndrome, and to what degree is this relationship sequentially mediated by creative and critical thinking? Addressing this question is important for both theoretical and practical reasons. Theoretically, the study advances understanding of the cognitive architecture underlying academic self-evaluations. Practically, it may inform the design of educational interventions aimed at fostering adaptive thinking patterns and reducing maladaptive self-doubt among university students. Based on the conceptual framework and the existing empirical literature, the following hypotheses were proposed:

*H1*: Computational thinking is positively associated with creative thinking.

*H2*: Computational thinking is positively associated with critical thinking.

*H3*: Creative thinking is positively associated with critical thinking.

*H4*: Creative thinking is negatively associated with academic imposter syndrome.

*H5*: Critical thinking is negatively associated with academic imposter syndrome.

*H6*: Computational thinking is negatively associated with academic imposter syndrome.

*H7*: Creative and critical thinking sequentially mediate the relationship between computational thinking and academic imposter syndrome.

By testing these hypotheses within a serial mediation framework, the present study aims to provide empirical evidence for a more comprehensive cognitive model of academic imposter syndrome. In doing so, it seeks to bridge the gap between higher-order cognitive skill development and psychological well-being in higher education, while also contributing to the development of evidence-based educational practices that support both intellectual and emotional resilience.

## 2 Materials and methods

### 2.1 Research design and study context

The present study employed a quantitative, cross-sectional survey design to examine the relationships among computational thinking, creative thinking, critical thinking, and academic imposter syndrome among undergraduate students (Creswell and Creswell, 2018). A cross-sectional design was considered appropriate because the study aimed to investigate associations among these variables within a natural educational setting rather than to establish causal effects. Data were collected during the [fall/spring] semester of the [2024–2025] academic year from undergraduate students enrolled in public universities in Türkiye. The study was conducted in regular instructional settings and focused on students from multiple disciplinary backgrounds in order to capture variation in academic experiences and cognitive dispositions.

### 2.2 Participants

**Figure 1:**
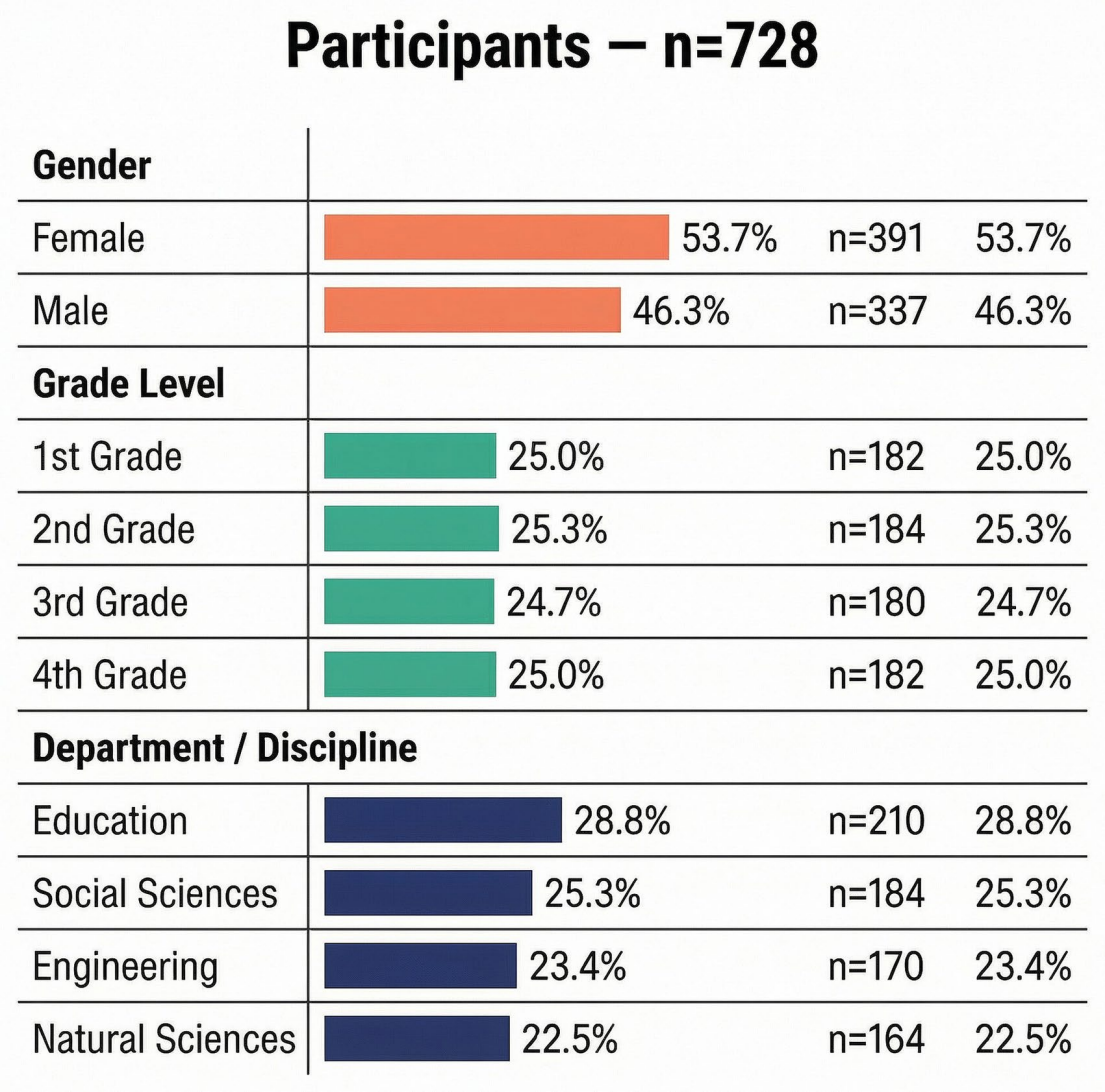
Participants.

A total of 728 undergraduate students voluntarily participated in the study. As shown in Figure 1, the sample consisted of 391 female students (53.7%) and 337 male students (46.3%). The distribution across grade levels was relatively balanced, including 182 first-year students (25.0%), 184 s-year students (25.3%), 180 third-year students (24.7%), and 182 fourth-year students (25.0%). In terms of academic discipline, 210 participants were enrolled in education programs (28.8%), 184 in social sciences (25.3%), 170 in engineering (23.4%), and 164 in natural sciences (22.5%). Participants ranged in age from 18 to 26 years, with a mean age of approximately 21 years. All participants were full-time students enrolled in public universities in Türkiye at the time of data collection. Students were recruited using convenience sampling through course announcements and institutional communication channels. Participation was entirely voluntary, and no academic or financial incentives were provided. The relatively balanced distribution across grade levels and the inclusion of students from multiple academic disciplines contributed to a heterogeneous sample structure, allowing the study to capture variation in cognitive and academic self-perceptions across different segments of the undergraduate population.

### 2.3 Measures

Four standardized measurement instruments were used to assess the main variables of the study. All scales were administered in their validated Turkish versions. Prior to the main analyses, internal consistency and construct validity of each instrument were re-examined within the present sample. Because the study relied on validated self-report instruments, the measured constructs should be interpreted as students’ reported dispositions, perceptions, and self-evaluative tendencies rather than as direct indicators of objective cognitive performance.

#### 2.3.1 Computational Thinking Scale (CTS)

Computational thinking was assessed using the Computational Thinking Scale (CTS) developed by Akyol (2020). The instrument consists of 30 items across three dimensions: Computational Thinking, Robotics-Coding and Programming, and Professional Development and Career Planning. Items are rated on a five-point Likert-type scale ranging from 1 (strongly disagree) to 5 (strongly agree), with higher scores indicating stronger computational thinking skills. Evidence from the original scale development study supported its construct validity through confirmatory factor analysis, and the reported reliability coefficients for the three subdimensions were 0.920, 0.843, and 0.884, respectively, with an overall reliability coefficient of 0.860. In the present study, the validated Turkish version of the scale was used. A representative item is: “I can solve the problems I encounter more systematically through computational thinking skills.” Accordingly, the CTS scores in the present study should be interpreted as self-reported computational thinking tendencies that may partly reflect applied technological engagement, rather than as purely performance-based indicators of abstract cognitive processing.

#### 2.3.2 Marmara Creative Thinking Dispositions Scale (MCTDS)

Creative thinking dispositions were assessed using the Marmara Creative Thinking Dispositions Scale (MCTDS) developed by Ozgenel and Cetin (2017). The scale consists of 25 items across six dimensions: innovation seeking, courage, self-discipline, inquisitiveness, skepticism, and cognitive flexibility. Responses are rated on a five-point frequency scale ranging from never to always, and total scores range from 25 to 125, with higher scores indicating stronger creative thinking dispositions. Previous validation studies have provided satisfactory evidence for the reliability and validity of the instrument. In the present study, the validated Turkish version of the scale was used. A representative item is: “I generate useful and original responses or solution pathways for problems or situations.”

#### 2.3.3 Marmara Critical Thinking Dispositions Scale (MCRTLDS)

Critical thinking dispositions were measured using the Marmara Critical Thinking Dispositions Scale (MCRTLDS) developed by Özgenel and Çetin (2018). The instrument consists of 28 items across six subdimensions: reasoning, judgment, evidence seeking, truth seeking, open-mindedness, and systematicity. Items are rated on a five-point Likert-type scale, with higher scores indicating stronger tendencies toward reflective and analytical thinking. The original validation study reported satisfactory evidence of reliability and validity, supporting the use of the scale in educational settings. In the present study, the validated Turkish version of the instrument was used. A representative item is: “I support my ideas with reliable information and strong evidence.” Similarly, the MCTDS and MCRTLDS were used to assess reported dispositions toward creative and critical thinking, not students’ objectively observed cognitive performance in these domains.

#### 2.3.4 Imposter Syndrome Scale (ISS)

Academic imposter-related perceptions were measured using the Academic Well-Being, Academic Incompetence, and Imposter Syndrome Scale developed by Ayyıldız et al. (2024). The instrument was developed based on data collected from undergraduate students enrolled at various universities in Türkiye and was designed to assess students’ academic self-perceptions across both positive and negative dimensions. The full scale consists of 37 items rated on a five-point Likert-type scale and is organized into three dimensions: Academic Well-Being (Items 1–9), Academic Incompetence (Items 10–21), and Imposter Syndrome (Items 22–37). The original development study reported strong psychometric properties for the instrument. Exploratory factor analysis yielded factor loadings ranging from 0.469 to 0.882, a Kaiser–Meyer–Olkin (KMO) value of 0.912, and a total explained variance of 67.32%. Internal consistency analysis produced a Cronbach’s alpha coefficient of 0.913, indicating high reliability. In addition, confirmatory factor analysis supported the factorial validity of the scale and yielded satisfactory model fit indices (*χ*^2^/*df* = 1.36, RMSEA = 0.04, NNFI = 0.96, SRMR = 0.03, AGFI = 0.92). These findings indicate that the scale has adequate validity and reliability for assessing university students’ academic well-being, perceived academic incompetence, and imposter-related self-perceptions. In the present study, the full scale was used as a composite measure of students’ academic imposter-related self-perceptions. To ensure interpretive consistency across the three dimensions, the Academic Well-Being items were reverse-coded before computing the total score. Thus, higher total scores indicated stronger academic self-doubt, higher perceived academic incompetence, and more pronounced imposter-related perceptions. A representative item from the Imposter Syndrome dimension is: “Fear of being ‘unmasked’ as a fraud in academic contexts.”

### 2.4 Procedure and data collection

Prior to data collection, ethical approval was obtained from the relevant institutional ethics committee. After ethical approval had been granted, the researchers informed potential participants about the purpose of the study, the voluntary nature of participation, confidentiality procedures, and their right to withdraw from the study at any time without penalty. Informed consent was obtained from all participants before the administration of the questionnaire. Data were collected during the [fall/spring] semester of the [2024–2025] academic year from undergraduate students enrolled in public universities in Türkiye. Participants were recruited through course announcements and institutional communication channels. The questionnaire package included a demographic information form and four standardized measurement instruments assessing computational thinking, creative thinking, critical thinking, and academic imposter syndrome. The survey was administered [using a mixed-mode procedure], and participants completed the questionnaire individually in approximately 20–25 min. To ensure anonymity and confidentiality, no personally identifiable information was collected. All responses were stored securely and were accessible only to the research team. The collected data were used solely for scientific purposes.

### 2.5 Data screening and preliminary analyses

Before conducting the main analyses, the dataset was screened for missing values, outliers, and distributional assumptions. Missing data were examined using descriptive statistics and were found to be minimal. Cases with excessive missing responses were excluded from further analyses. Univariate and multivariate outliers were evaluated using standardized scores and Mahalanobis distance (Tabachnick and Fidell, 2019). Normality assumptions were assessed through skewness and kurtosis values, as well as visual inspection of histograms and Q–Q plots. All variables demonstrated acceptable levels of normal distribution. Multicollinearity was examined using variance inflation factor (VIF) and tolerance values, which indicated no serious multicollinearity problems. In addition, common method bias was evaluated using Harman’s single-factor test, and no substantial bias was detected (Podsakoff et al., 2003). Nevertheless, Harman’s single-factor test should be regarded as an initial diagnostic only, and it does not completely rule out the possibility of common method variance.

### 2.6 Data analysis strategy

Data analyses were conducted using IBM SPSS Statistics and the PROCESS macro (Version 4.0). Descriptive statistics and Pearson correlation coefficients were calculated for all study variables. Internal consistency reliability was assessed using Cronbach’s alpha and McDonald’s omega coefficients (Dunn et al., 2014; McDonald, 1999). To test the proposed serial mediation model, Hayes’ PROCESS Model 6 was employed (Hayes, 2018). In this model, computational thinking was specified as the independent variable, creative thinking as the first mediator, critical thinking as the second mediator, and academic imposter syndrome as the dependent variable. Bootstrap resampling with 5,000 samples was applied to estimate indirect effects and generate 95% bias-corrected confidence intervals. Indirect effects were considered statistically significant when the confidence interval did not include zero. Gender, age, and academic grade level were included as control variables in the mediation analyses to account for potential confounding effects. Effect sizes and standardized regression coefficients were reported to facilitate interpretation of the findings.

### 2.7 Ethical considerations

The study was conducted in accordance with the ethical standards of the Declaration of Helsinki and relevant institutional guidelines. Ethical approval was obtained from the university ethics committee prior to data collection. All participants provided informed consent and were assured of confidentiality and anonymity. Participation was voluntary, and no form of coercion was involved. Data were used solely for scientific purposes and were stored securely throughout the research process.

## 3 Results

### 3.1 Preliminary analyses

Prior to the main data collection, all measurement instruments were subjected to a pilot application. The findings obtained from the validity and reliability analyses are summarized in Table 1.

**Table 1:** Validity and reliability analysis results.

Variable	Reliability (*α*)		Validity-CFA-original study				Validity-CFA-pilot study			
	Original	Pilot	*χ*^2^/df	RMSEA	SRMR	CFI	*χ*^2^/df	RMSEA	SRMR	CFI
CTS	0.860	0.867	1.81	0.03	0.04	0.98	2.11	0.04	0.05	0.95
MCTDS	0.878	0.866	2.07	0.05	0.05	0.90	2.34	0.05	0.04	0.89
MCRTLDS	0.910	0.902	—	—	—	—	2.47	0.03	0.04	0.96
ISS	0.912	0.928	1.36	0.04	0.03	0.93	1.59	0.05	0.04	0.92

*p* < 0.05; *α* Cronbach’s alpha; CFA Confirmatory factor analysis.

*χ*^2^/*df*-RMSEA-SRMR-CFI; Goodness-of-fit index for CFA analysis.

The results of the validity and reliability analyses for the measurement instruments are presented in Table 1. Internal consistency coefficients indicated satisfactory reliability for all scales in both the original and pilot studies. Cronbach’s alpha values ranged from 0.860 to 0.912 in the original samples and from 0.866 to 0.928 in the pilot samples, demonstrating high levels of internal consistency. Confirmatory factor analysis results provided further support for the construct validity of the instruments. For the original samples, the *χ*^2^/*df* ratios ranged from 1.36 to 2.07, RMSEA values varied between 0.03 and 0.05, SRMR values ranged from 0.03 to 0.05, and CFI values ranged from 0.90 to 0.98. These indices indicate acceptable to excellent model fit. Similarly, CFA results obtained from the pilot study demonstrated satisfactory fit, with *χ*^2^/*df* values ranging from 1.59 to 2.47, RMSEA values between 0.03 and 0.05, SRMR values between 0.04 and 0.05, and CFI values between 0.89 and 0.96. Overall, the findings indicate that all measurement instruments exhibited strong internal consistency and satisfactory factorial validity across both datasets. The consistency of reliability and model fit indices between the original and pilot samples suggests that the instruments functioned reliably and maintained stable psychometric properties. Therefore, all scales were considered appropriate for use in the subsequent analyses. Following the establishment of reliability and construct validity, descriptive statistics and intercorrelations among the study variables were examined.

Descriptive statistics and intercorrelations among the study variables are presented in Table 2. The results indicated that all variables were significantly correlated at the 0.01 level. Computational thinking was positively associated with creative thinking (*r* = 0.52, *p* < 0.01) and critical thinking (*r* = 0.49, *p* < 0.01). Similarly, creative thinking was positively associated with critical thinking (*r* = 0.62, *p* < 0.01). In contrast, academic imposter syndrome was negatively associated with computational thinking (*r* = −0.64, *p* < 0.01), creative thinking (*r* = −0.55, *p* < 0.01), and critical thinking (*r* = −0.69, *p* < 0.01). These findings indicate that higher levels of computational, creative, and critical thinking were associated with lower levels of imposter-related perceptions. The observed correlation coefficients suggest moderate to strong relationships among the study variables and provide preliminary support for the proposed mediation model. In addition, skewness and kurtosis values were within acceptable ranges, suggesting that the distributions of the variables approximated normality. Internal consistency coefficients were also high across all measures (*α* = 0.866 to 0.928), supporting the reliability of the data for subsequent mediation analyses.

**Table 2:** Descriptive statistics and intercorrelations among study variables.

Variable	1 [95% CI]	2 [95% CI]	3 [95% CI]	4 [95% CI]
1. CTS	—			
2. MCTDS	0.52** [0.42, 0.58]	—		
3. MCRTLDS	0.49** [0.44, 0.61]	0.62** [0.52, 0.66]	—	
4. ISS	−0.64 [−0.71, −0.59]	−0.55 [−0.62, −0.50]	−0.69 [−0.74, −60]	—
*α*	0.867	0.866	0.902	0.928
*M*	135.52	109.28	126.74	164.26
SD	5.62	3.92	7.14	9.27
Skewness	0.075	0.342	0.101	0.124
Kurtosis	0.523	0.496	0.269	0.425

**p < 0.01; *α* Cronbach’s alpha; M, mean; SD, standard deviation.

Computational Thinking Scale (CTS); Marmara Creative Thinking Dispositions Scale (MCTDS).

Marmara Critical Thinking Dispositions Scale (MCRTLDS); Imposter Syndrome Scale (ISS).

### 3.2 Serial mediation analysis results

To examine the proposed serial mediation model, a series of analyses were conducted using PROCESS Model 6, and the results are presented in Table 3.

**Table 3:** Serial mediating roles of creative and critical thinking in the relationship between computational thinking and imposter syndrome.

Panel A. direct effects	Hypotheses	Coefficient (*b*)	*p*	95% CI		Results
				LL	UL	
CTS → MCTDS	H1	0.48	<0.001	0.40	0.56	Supported
CTS → MCRTLDS	H2	0.41	<0.001	0.31	0.51	Supported
MCTDS→MCRTLDS	H3	0.46	<0.001	0.36	0.58	Supported
MCTDS→ISS	H4	−0.28	<0.001	−0.40	−0.16	Supported
MCRTLDS→ISS	H5	−0.34	<0.001	−0.48	−0.20	Supported
CTS → ISS	H6	−0.22	<0.001	−0.32	−0.12	Supported
Total effect (CTS → ISS)	—	−0.44	<0.001	−0.54	−0.34	—
Direct effect (CTS → ISS, controlling for mediators)	—	−0.22	<0.001	−0.32	−0.12	—

Panel B. indirect effects	Related hypothesis	Effect	BootSE	BootLLCI	BootULCI	Result
CTS → MCTDS → ISS	H7	−0.095	0.019	−0.136	−0.063	Supported
CTS → MCRTLDS → ISS	H7	−0.068	0.015	−0.100	−0.043	Supported
CTS → MCTDS → MCRTLDS → ISS	H7	−0.057	0.012	−0.082	−0.037	Supported
Total indirect effect	H7	−0.220	0.041	−0.310	−0.150	Supported

CI, Confidence interval; LL, lower limit; UL, upper limit; Bootstrap = 5,000 samples.

Computational Thinking Scale (CTS); Marmara Creative Thinking Dispositions Scale (MCTDS).

Marmara Critical Thinking Dispositions Scale (MCRTLDS); Imposter Syndrome Scale (ISS).

**Figure 2:**
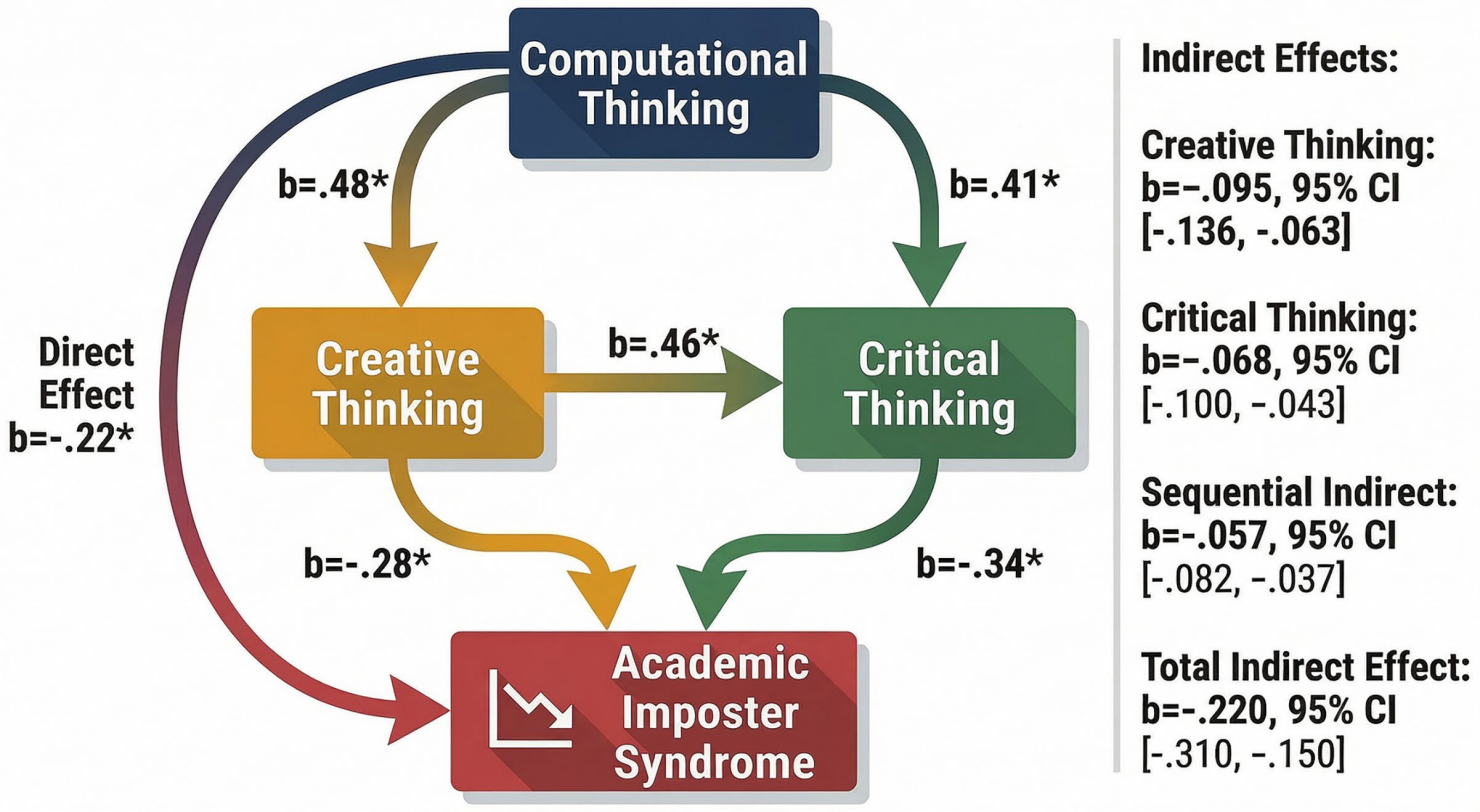
Visualization of serial mediation effects in the proposed model.

The results of the serial mediation analysis are presented in Table 3. Consistent with the proposed hypotheses, computational thinking was positively associated with creative thinking (*b* = 0.48, *p* < 0.001) and critical thinking (*b* = 0.41, *p* < 0.001), supporting H1 and H2. In addition, creative thinking significantly and positively predicted critical thinking (*b* = 0.46, *p* < 0.001), providing support for H3. Regarding the outcome variable, both creative thinking (*b* = −0.28, *p* < 0.001) and critical thinking (*b* = −0.34, *p* < 0.001) were negatively associated with academic imposter syndrome, supporting H4 and H5. Computational thinking also demonstrated a significant direct negative effect on academic imposter syndrome (*b* = −0.22, *p* < 0.001), confirming H6. Bootstrap analyses further revealed significant indirect effects of computational thinking on academic imposter syndrome through creative thinking (*b* = −0.095, 95% CI [−0.136, −0.063]) and through critical thinking (*b* = −0.068, 95% CI [−0.100, −0.043]). In addition, the sequential indirect pathway through creative thinking and critical thinking was also significant (*b* = −0.057, 95% CI [−0.082, −0.037]). Because none of the confidence intervals included zero, the indirect effects collectively supported H7. The total indirect effect was also significant (*b* = −0.220, 95% CI [−0.310, −0.150]). Finally, the total effect of computational thinking on academic imposter syndrome was significant (*b* = −0.44, *p* < 0.001), while the direct effect remained significant after controlling for the mediators (*b* = −0.22, *p* < 0.001). These findings indicate partial mediation, suggesting that creative thinking and critical thinking partially explain the relationship between computational thinking and academic imposter syndrome. To further illustrate the magnitude of direct and indirect effects identified in the mediation analysis, these relationships are visually presented in Figure 2.

## 4 Discussion and conclusion

This study examined the relationships among computational thinking, creative thinking, critical thinking, and academic imposter syndrome within a serial mediation framework. By integrating cognitive and psychological perspectives, the study provides a more comprehensive account of how higher-order thinking skills are related to students’ academic self-perceptions and emotional well-being. In this respect, the findings extend previous work emphasizing the educational and psychological relevance of computational and higher-order thinking in higher education settings (Günaltay et al., 2025; Habib et al., 2024; Montuori et al., 2024; Sun et al., 2023). Before interpreting the substantive findings, it is important to note that the measurement instruments demonstrated satisfactory psychometric properties. The reliability and validity findings were consistent with previous validation studies, suggesting that the scales adequately captured the intended constructs of computational thinking, creative thinking, critical thinking, and academic imposter syndrome (Akyol, 2020; Ayyıldız et al., 2024; Özgenel and Çetin, 2018). This point is particularly important because mediation findings can be meaningfully interpreted only when the underlying measurement tools are sufficiently reliable and valid (Dunn et al., 2014; Hayes, 2018). The correlation findings showed that computational thinking was positively associated with creative and critical thinking, whereas these cognitive variables were negatively associated with academic imposter syndrome. This pattern is in line with perspectives suggesting that analytical reasoning, creative problem-solving, and metacognitive regulation operate in an interconnected manner in students’ academic functioning (Li et al., 2024; Montuori et al., 2024; Wu et al., 2025; Yao and Lin, 2025). It also supports the argument that students who can approach academic tasks in a more systematic and flexible way may be better able to regulate their responses to academic difficulty and uncertainty (Dörrenbächer-Ulrich et al., 2024; Hemmler and Ifenthaler, 2024).

The regression results further demonstrated that computational thinking significantly predicted both creative and critical thinking. This finding supports the view that computational thinking is more than a technical or discipline-specific skill; rather, it appears to function as a foundational cognitive competence involving abstraction, decomposition, and structured reasoning (Denning, 2017; Wing, 2006). In line with earlier studies, repeated engagement in computational problem-solving may cultivate habits of mind that support innovation, reflection, persistence, and analytical rigor (Günaltay et al., 2025; Hong et al., 2025; Shute et al., 2017; Sun et al., 2023; Yao and Lin, 2025). The positive association between creative and critical thinking also deserves attention. While creativity allows students to generate novel ideas and explore alternative perspectives, critical thinking provides the evaluative and reflective framework needed to assess the quality and feasibility of these ideas (Kenett, 2025; Runco and Jaeger, 2012). In this sense, the present findings reinforce the view that creativity and critical thinking are not competing processes but mutually supportive dimensions of higher-order cognition. This interpretation is consistent with recent work highlighting the close relationship between idea generation, reflective judgment, and academic engagement (Shaber et al., 2025; Villalustre et al., 2024). In relation to the outcome variable, both creative thinking and critical thinking emerged as significant negative predictors of academic imposter syndrome. This finding is consistent with prior research showing that stronger cognitive self-regulation, more realistic self-evaluation, and more adaptive academic self-concepts are associated with lower levels of impostor-related experiences (Chua et al., 2025; Ménard and Chittle, 2023; Yang et al., 2024). Students who are able to evaluate their performance more accurately and approach academic challenges more flexibly may be less likely to attribute their achievements to luck or to perceive themselves as intellectually inadequate (Dumitrescu and De Caluwé, 2024; Jansson et al., 2025). The direct negative effect of computational thinking on academic imposter syndrome further highlights the broader psychological relevance of structured reasoning skills. Students with stronger computational thinking abilities may be more likely to perceive academic challenges as manageable, organized, and solvable rather than ambiguous or threatening. This may reduce uncertainty, anxiety, and self-doubt, and may contribute to a stronger sense of academic competence. Similar interpretations can be found in studies linking problem-solving competence, reflective thinking, and academic self-efficacy to more adaptive academic self-perceptions (Borbon et al., 2025; Hartelt and Martens, 2024).

Most importantly, the serial mediation analysis showed that creative and critical thinking sequentially mediated the relationship between computational thinking and academic imposter syndrome. This finding suggests that the effect of computational thinking is not limited to its direct contribution to task management or academic reasoning. Instead, computational thinking appears to influence students’ academic self-perceptions partly by strengthening intermediate cognitive processes, particularly flexible idea generation and reflective evaluation. In this respect, the present findings are consistent with studies emphasizing the importance of integrated cognitive processes in students’ learning and self-regulation (Hsu et al., 2024; Michel-Villarreal et al., 2023; Para et al., 2024). However, these findings should be interpreted as associations consistent with the proposed model rather than as definitive evidence of temporal or mechanistic causality. This result also aligns with contemporary models of academic functioning that emphasize the cumulative and interconnected effects of multiple cognitive competencies rather than isolated skills (Anderson, 2015; OECD, 2019). Rather than exerting separate influences, computational thinking, creative thinking, and critical thinking seem to operate within a broader cognitive system that shapes both academic performance and emotional adjustment (Hemmler and Ifenthaler, 2024). Accordingly, the present findings suggest that imposter-related academic self-perceptions may be better understood within a multidimensional cognitive framework. From a theoretical perspective, the current findings contribute to the literature by supporting a more integrated approach to higher-order thinking. Previous research has often examined computational thinking, creativity, and critical thinking independently, especially in relation to achievement, digital skills, or general learning outcomes (Ngadengon et al., 2024; Ocampo et al., 2024). In contrast, the present study shows that these constructs may operate in a more dynamic and hierarchical way, jointly contributing to students’ academic self-perceptions. In this sense, the findings also support developmental perspectives suggesting that higher-order thinking skills do not emerge in isolation, but through interrelated and progressive cognitive processes.

The study further suggests that computational thinking may function as an initial cognitive foundation in this process. By helping students organize information, identify patterns, and apply systematic strategies, computational thinking may facilitate both creative exploration and critical evaluation (Montuori et al., 2024; Yao and Lin, 2025). This interpretation extends earlier research by empirically supporting the sequential structure of cognitive skill development in higher education. From a pedagogical standpoint, the findings underscore the importance of designing learning environments that promote computational, creative, and critical thinking simultaneously. Instructional approaches such as project-based learning, inquiry-based learning, and interdisciplinary problem-solving may be especially useful in this regard (Hague, 2024; Hong et al., 2025; Wu et al., 2025; Zaremohzzabieh et al., 2025). Such approaches may support not only academic engagement and reflective learning, but also students’ resilience against maladaptive academic self-beliefs. The results also have implications for teacher education and professional development. Teachers play an important role in shaping students’ cognitive and emotional experiences. Educators who are trained to promote higher-order thinking may help create learning environments that are more supportive, reflective, and empowering (Hartelt and Martens, 2024; Rajadurai and Ganapathy, 2023). In this sense, integrating computational and creative problem-solving into teacher education programs may contribute indirectly to students’ psychological well-being. At the institutional level, universities may benefit from incorporating cognitive skill development into broader student support frameworks (Dumitrescu and De Caluwé, 2024; Para et al., 2024). Workshops and structured activities focusing on computational reasoning, creative inquiry, and reflective evaluation may complement traditional counseling services (Emas et al., 2024; Pérez-Jorge et al., 2025). Such efforts may help students build more resilient academic identities and reduce vulnerability to imposter-related experiences. Despite these contributions, the study has several limitations. First, the cross-sectional design does not allow causal interpretations. Second, the reliance on self-report measures may have introduced response biases. More specifically, the observed relationships reflect students’ reported dispositions, perceptions, and self-evaluations rather than objectively assessed cognitive performance. For this reason, some portion of the observed associations may be attributable to shared method variance and self-perception bias. Third, because the sample was drawn from Turkish universities, the findings should be generalized with caution to other educational and cultural settings. In addition, although gender, age, and academic grade level were statistically controlled, academic discipline was not included as a covariate; therefore, possible disciplinary variation in the observed relationships should be interpreted with caution. Future research would benefit from longitudinal and experimental designs, the inclusion of performance-based or observational indicators, and cross-cultural comparisons. Future studies may also extend the present model by incorporating related psychological variables such as academic self-efficacy, resilience, and learning motivation (Newar et al., 2025). Such variables may function as additional mediators or moderators and may help explain why some students are more vulnerable to imposter-related experiences than others. Moreover, intervention-based studies examining whether targeted cognitive training can reduce academic imposter syndrome would provide practical insights for both educators and institutions (Para et al., 2024).

In conclusion, the present study shows that computational thinking is positively linked to creative and critical thinking and negatively linked to academic imposter syndrome. More importantly, creative and critical thinking partially and sequentially mediate this relationship. These findings support a more holistic understanding of higher-order thinking in higher education and suggest that the development of cognitive skills may also contribute to healthier and more adaptive academic self-perceptions. At the same time, this conclusion should be understood in relation to self-reported higher-order thinking tendencies and academic self-perceptions rather than directly observed cognitive performance.

## 5 Implications

The findings of the present study have implications for theory, educational practice, institutional policy, and future research. From a theoretical perspective, the results support a hierarchical and interconnected model of higher-order thinking, showing that computational thinking may function as a foundational competence that facilitates the development of creative and critical thinking. In this respect, the study extends existing frameworks by demonstrating how multiple cognitive processes may interact sequentially to shape students’ academic self-perceptions and emotional well-being.

From an educational perspective, the findings suggest that higher education curricula should embed computational, creative, and critical thinking in an integrated manner rather than treating them as isolated outcomes. Instructional practices such as project-based learning, problem-based learning, coding-integrated activities, inquiry-based tasks, and interdisciplinary learning experiences may help promote these competencies while also reducing students’ vulnerability to academic imposter syndrome (Hague, 2024; Hong et al., 2025; Wu et al., 2025; Zaremohzzabieh et al., 2025). Teacher education programs may benefit especially from structured opportunities for future teachers to develop computational and creative problem-solving capacities, as such preparation may contribute to more supportive classroom climates and stronger academic self-confidence among learners (Hartelt and Martens, 2024; Rajadurai and Ganapathy, 2023).

At the institutional level, universities may consider integrating cognitive skill development into advising, mentoring, and counseling services. Workshops focusing on problem-solving, reflective thinking, and creative inquiry may complement traditional student support structures and help students develop more resilient academic identities (Dumitrescu and De Caluwé, 2024; Emas et al., 2024; Para et al., 2024; Pérez-Jorge et al., 2025). Finally, future research should continue to examine the developmental trajectories of computational, creative, and critical thinking and their relationship with academic imposter syndrome. Longitudinal, experimental, cross-cultural, and mixed-method designs would be especially useful, as would studies incorporating additional variables such as self-efficacy, motivation, and resilience (Newar et al., 2025; Para et al., 2024).

## Data Availability

The data analyzed in this study is subject to the following licenses/restrictions: The authors will provide the original data underlying the findings of this study upon reasonable request, without any restrictions. Requests to access these datasets should be directed to AY, yilmazadem@kastamonu.edu.tr.
